# The Late Architectural Craze

**Published:** 1882-10

**Authors:** Ben. H. Pratt


### THE BISTOURY.

Vol. ~~XIX~~. XVIII ELMIRA, N. Y., OCTOBER, 1882. No. 3.

### Contributions.

### THE LATE ARCHITECTURAL CRAZE.

BEN H. PRATT.

There has come into vogue a style of domestic architecture which is of a type so pronounced as to compel attention, extort criticism and almost ostracize the taste that declines to endorse it.

It is no more nor less than an architectural craze, known to the technical as the Queen Ann style; to others as the composite, miscellaneous, conservative, esthetic, early English, anti-conventional, and by a hundred other names, appropriate or otherwise.

By whatever name we know it, it is simply that style which allows him who adopts it to do precisely what he pleases, consistent with possibilities, regardless of what one ever has done or ever expects to do. It is literally the go-as-you-please style of architecture; then why not so call it at once?

There was doubtless very good reason for this new departure in our system of home building, for such it almost exclusively is, since the style partakes too much of the individuality of the person or persons for whom it is adopted, to be particularly applied to any other class of buildings. Hence it is not a style appropriate for tenement use, nor could it be applied to any of the uses for which public buildings, such as churches, hotels, business blocks, government, state or county buildings are put. The innovation sprang from a long felt tendency to cut loose from a certain set form of domestic architecture which had well nigh become a *sine qua non*, to violate which was to isolate one's self from the conventional, and make one conspicuously different from the multitude. It was born of desperation, as it were. Architectural conventionalism had begun to exercise a sort of arbitrary rule, and its governance was warping, distasteful, biasing, confining, discommoding. That its dictatorial sway has been completely usurped, no one who examines into the new style can for a moment doubt. The monocracy has ceased to be. Now, no two may build according to one's dicta. So far, the spirit is admirable. Had the reform not resulted in a revolution more complete than this, it would have been a laudable one. That it can scarcely be so designated under the loose rein given, it will appear as we go on.

The reform was needed. The temptation to break away from the old *regime* was almost resistless. The severity of the preceding style was slavery. Our homes were becoming nothing if not stately, even palatial. Elegance, lavish expenditure, grandeur, were usurping simplicity, comfort, convenience, utility. The result was a residence, an abode, family headquarters—not a home. The family, removed to its new place, found itself much like a rare specimen of something enclosed in a show case. Things were too nice to use, too delicate to handle. There was a Sunday-all-the-week atmosphere pervading it, that stifled its inmates and repelled its visitors. Exactitudes, equipoises, things to match, uniformities, were painful characteristics of the old *regime.* It is not surprising that rebellion ensued, but it is astonishing how fierce the reaction has become.

Seized on its way by the ultraist and experimentalist, the new departure has been carried beyond the pale of common sense and creature comforts into the realm of caricature, and already the idea seems to have become, not how we may make our homes absolutely comfortable and convenient by disregarding all architectural law as hitherto interpreted, but how far we may go in the direction of the quaint and the queer and the *outre*, regardless of comfort and every thing except to have nothing as people would naturally expect to find it. This is the bad in the new craze.

We Americans are the most violent extremists in the world. We can do nothing in moderation. Ultraism is our besetting fault. Had the new departure been taken up in the spirit of moderation; had we determined to make our homes comfortable, and adapted to all the purposes of living, regardless of looks or of opinions, and stopped there, the change would have been hailed as the blessing of the age—the domestic achievement of the century; but to make the home singular for the sake of the singularity, and to strive merely to see how odd and abnormal and monstrous we could design and construct something to call a home, is not only absurd, but stamps such builders as a lot of cranks.

But the novelty was too much for us. Every body with an excuse to build immediately began to rack his brain to discover how uncouth a design for a dwelling place might lurk in his imagination. If he couldn't find any thing in his own head sufficiently abortive to outrage every thing that had yet been produced, he would call upon his most cranky friends to help him to some startling nondescript of a notion to further remove his proposed dwelling from the likeness of any thing in the heavens above, in the earth beneath, or in the waters under the earth. Those with homes builded before the craze came, sought out how they might reconstruct or alter them into something equally stunning.

Wide as the doors of opportunity had been opened to the genius and ingenuity of men in the simple direction of primitivism, comfort, convenience and taste, they were not content to take that course. What an admirable chance was given for the exercise of individuality, limited only by the household's needs! The release from conventionalism was complete. None need be under the slightest restraint because of what another might think or say. He had the full tether of his tastes and inclinations, and the opportunity to show all who came his ideal of a complete dwelling spot, whether expensive or plain. It was his opportunity to reveal the breadth of his idiosyncrasy, but he made it only the measure of the height of his folly, or the depth of his ignorance, or the narrowness of his ambition, or the littleness of his genius. With the opportunity of the wise came the chance of the crank. We have the results of both all around us. We find inviting simplicity and refinement of taste and purity of design in many of the new homes; but in many more we find the all too bold searching after startling effects, coarse application and misuse of a borrowed conceit, and maladaptations that stamp those who placed them as marvels of ignorance. The one class is honestly in search of such a combination of utility, comfort, health, convenience and beauty as will blend into a symmetrical abode; the other class has gathered together all the surprises and oddities at their command and arranged them into a junk-shop. One has a home and the other a museum.

There is no special merit in being simply odd. To strive to be odd is an affectation inexcusable. Where one's oddness works no inconsistency, it is often attractive and sometimes pleasing. If a man choose to build his dwelling all on the ground floor, constructing in the center of his house one big living room, sky-lighted, forty feet high, galleried as to one end, and surrounding it with drawing-room, sitting-room, library, dormitories, lavatories, and other apartments opening directly out of it, he is odd, but simple; or if he put his bed-rooms on the first floor and his kitchen on the second, he is odd, but wise; or if he banish carpets and other moth-harboring haberdashery, and introduce bare floors, he is odd, but likely a healthy and a scrupulously clean man; or if he prefers an eight to a fourteen or sixteen foot ceiling, he may be queer but he is humane to those that must often mount stairs or carry coals; or if one locate his front door at the rear of the side of his house and thus brings you into a corridor that compels you to go twice over the distance necessary to reach his living room, he is odd, but unreasonable; or if one construct a pretty balcony and leave no means to gain access to it, he is odd, but inconsistent; or if one put a big window where a small one is better, or a small one where a big one is requisite, proves himself odd, but a fool; or if one build of stone a little way, of brick a little way, of tile a little way, of slate a little way, of clapboards a little way, of shingles a little way, of rough-cast a little way, and otherwise seek to compel attention to his oddness, he is but an ostentatious crank whom nature should have better designated by long and hairy ears.

Verily the new architectural craze has revealed the character, the peculiarity, the individuality, the wisdom, the folly, the vanity, the conceit, the arrogance, the ingenuity, the humanity, the churlishness, the vulgarity, the shoddiness, the idiosyncrasy, the pride, the presumption, the nobility, the envy, the intellect, the refinement, the taste, the discernment, the discretion, the skill, the knack, the sagacity, the levity, the weakness, the egotism—in fact about all the good, bad and indifferent characteristics of the human mind as no other innovation that the age has thrust upon us has succeeded in doing.

Scarcely a town or village with any pretensions whatever to enterprise but presents both classes of the new order of newly erected dwellings. Some are mere monstrosities of bastard architecture, a conglomeration of architectural whims, huddled together with no evidence of purpose other than to see how great a curiosity can be devised under the guise of a possible home. Inconsistencies stare upon the visitor on every hand. Incongruities, contradictions, maladaptations reveal the designer's ignorance and boorishness on all sides. One loses patience with, and is inspired with disgust for, the thing and the thing maker, and wonders which imp of the *inferno* did *Diabolus* make to squat at the designer's ear and whisper therein such atrocious violations of every recognized ingredient in architectural harmony.

On the other hand, we find in many the pleasing results aimed at by the projectors of the new code architectural. The simplicities have been studied there with delicious effect. There are the odd and the quaint, but introduced without intrusiveness, seemingly belonging there because no shock nor jar comes of their presence. They settle down upon and give an impress to the whole which affords exquisite pleasure. There is a nice adaptation of the obsolete, the antique, the disused, the old fashioned and the primitive, to the new, recent, modern. The recondite does not seem far-fetched nor whimsical, but ingenuous as well as ingenious, and in no sense disturbing. Perversions do not strike the observer as such. Odd things do not seem unnatural, so nice are the adjustments. There is no hint of affectation, for all displays a purpose which does not require a commentary in explanation of what is revealed. We rather feel than see how every revival of the quaint came there and belongs there. Comfort, convenience, health, beauty, harmony, are combined with that rare sense which implies breadth of purpose and a well balanced taste. It is, *par excellence*, a home in every sense; such a home as would beget no mental distortions, but prove a constant educator and refiner of the young and a singular solace for the maturer mind; such a home as would prove a perennial source of quiet pleasure for all within it, never wearying, never startling, ever gently impressing and elevating, like beatitudes daily renewed. For giving us such homes we bless the new departure, and while we can not in too strong terms deprecate the abuse of the innovation, we have faith enough in the evidently deepening and broadening taste of this generation to believe that good will so far overshadow the evil tendencies of the new departure that the results will be most happily apparent in our domestic relations, and not only redound to the elevation of a legitimate taste in architecture merely, but prove the initial step in entering that broader field of esthetic culture of which we have as yet had but a glimpse through the medium of sentimentalism and its caricaturing exemplars and exponents.

The quaint homes rising around us are giving, withal, a picturesqueness to our resident squares that has never before been approached. The forbidding, uninviting, repellant piles of brick and stone, and the wooden boxes, window perforated, that we have heretofore been called upon to point out as homes, are, happily, to become *passe*, and while it may be many years before the severities of their exteriors may disappear before the better taste of coming inmates, we believe the time is not far distant when the stately will be as infrequent in our homes as are to-day the pretty, quaint, multi-gabled, low-squat cottages which are the true exponents of the late architectural craze.

